# Topological principles and developmental algorithms might refine diffusion tractography

**DOI:** 10.1007/s00429-018-1759-1

**Published:** 2018-09-27

**Authors:** Giorgio M. Innocenti, Tim B. Dyrby, Gabriel Girard, Etienne St-Onge, Jean-Philippe Thiran, Alessandro Daducci, Maxime Descoteaux

**Affiliations:** 1 Department of Neuroscience, Karolinska Institutet, Stockholm, Sweden; 2 Brain and Mind Institute, Ecole Polytechnique Féderale de Lausanne EPFL, Lausanne, Switzerland; 3 Signal Processing Laboratory (LT55) Ecole Polytechnique Féderale de Lausanne (EPFL-STI-IEL-LT55), Station 11, 1015 Lausanne, Switzerland; 4 Danish Research Centre for Magnetic Resonance, Center for Functional and Diagnostic Imaging and Research, Copenhagen University Hospital Hvidovre, Hvidovre, Denmark; 5 Department of Applied Mathematics and Computer Science, Technical University of Denmark, Kongens, Lyngby Denmark; 6 Sherbrooke Connectivity Imaging Laboratory (SCIL), Computer Science Department, Faculty of Science, Université de Sherbrooke, Quebec, Canada; 7 Computer Science Department, University of Verona, Verona, Italy; 8 Department of Nuclear Medicine and Radiobiology, Sherbrooke Molecular Imaging Center, Faculty of Medicine and Health Science, Université de Sherbrooke, Sherbrook, Canada; 9 Department of Radiology, University Hospital Center (CHUV), University of Lausanne (UNIL), Lausanne, Switzerland

**Keywords:** Diffusion MRI, Tractography, Axons, Brain pathways, Brain development

## Abstract

The identification and reconstruction of axonal pathways in the living brain or “ex-vivo” is promising a revolution in connectivity studies bridging the gap from animal to human neuroanatomy with extensions to brain structural–functional correlates. Unfortunately, the methods suffer from juvenile drawbacks. In this perspective paper we mention several computational and developmental principles, which might stimulate a new generation of algorithms and a discussion bridging the neuroimaging and neuroanatomy communities.

**Electronic supplementary material:**

The online version of this article (10.1007/s00429-018-1759-1) contains supplementary material, which is available to authorized users.

## Introduction

Brain sciences are undergoing a paradigm shift. After decades of attention to the organization and function of gray matter led by the recording of evoked potentials, single neurons activity, positron emission tomography (PET), functional magnetic resonance (fMRI), and by detailed analysis of local cortical connectivity, the focus is now shifting towards the white matter and the axons traveling therein. In the eighteenth century, M de la Peyronie, surgeon of Luis XV of France (1744), had suggested that the corpus callosum is the site “where the soul implements its functions”. That notion was controversial. However, the importance of the white matter in brain function was stressed by two influential papers: Geschwind’s “disconnexion syndromes” (1965a, b) and Sperry’s split-brain studies (1982). Clearly, lesions of cortico-cortical connections lead to deficits ranging from aphasia to agnosia while the involvement of the white matter might underlie pathologies ranging from dyslexia (Klingberg et al. 2000) to schizophrenia (Innocenti et al. 2003).

Neural connections were studied with a number of invasive techniques in animals, ranging from the visualization of degenerating fibers to axonal transport of molecules injected in the brain (Zaborszki et al. 2006). Among these, the retrograde transport of Horse Radish Peroxidase (HRP), introduced by Kristensson and Olson (1971), provided a very detailed, semi-quantitative picture of connections in a number of species, including the macaque monkey. The anterograde transport of biocytin or biotinilated dextran provided a detailed image of single axons, their terminal arbor geometry including the size and distribution of synaptic boutons (King et al. 1989; Innocenti and Caminiti 2017).

More recently, the study of anisotropic water diffusion with MRI and the development of diffusion tractography algorithms provided tools to visualize neural connections as “streamlines” each estimating a fascicle of axons, in the intact brain, including the human brain (Conturo et al. 1999; Mori et al. 1999; Basser et al. 2000; Mori and van Zijl 2002; Dauguet et al. 2007; Dyrby et al. 2011, 2018; Tournier et al. 2011; Jeurissen et al. 2017). Streamlines coursing together delineate bundles and several bundles correspond to tracts or fasciculi of classical histology, e.g. the corpus callosum, the corticospinal tract, the longitudinal fasciculi, etc. The potentials of this approach are enormous. First, the technique is non-invasive and translational between animals and humans (Innocenti et al. 2016, 2018; Safadi et al. 2018). Second, the identification of white matter pathways can be applied to the whole brain, is much faster than histology and, therefore, can be applied to groups of individuals of a given species. Finally, this technique could extend to the human what is known only in animals, in particular primates, and eventually, it could identify differences in neural connections associated with individual special skills as well as with as neurological and psychiatric syndromes.

It was authoritatively stated that diffusion tractography cannot achieve both high sensitivity and high specificity (Thomas et al. 2014; Knösche et al. 2015). Indeed, In spite of the several astute algorithms proposed to “clean” diffusion tractography (Sommer et al. 2016; Schurr et al. 2018 and references therein) the method still suffers from a number of drawbacks (Jones and Cercignani 2010; Jones et al. 2012; Daducci et al. 2016; Maier-Hein et al. 2017). One of these is the generation of false negatives, i.e. connections which are not identified. This is largely due to “hard-to-track” regions of the brain, which suffer from partial volume effects and poor resolution. This leads to difficulties in tracking narrow corridors of white matter particularly when axonal crossing occurs. Tractography algorithms using anatomical information from a high-resolution T1-weighted image have been proposed to guide tractography toward the gray matter and reduce bias in the narrow white matter pathways (Smith et al. 2012; Girard et al. 2014; Schurr et al. 2018). This has been shown to reduce some of the bias in the overall streamline reconstruction but other bias remains, such as streamlines neglecting the bank of sulci (Van Essen et al. 2014; Reveley et al. 2015; Donahue et al. 2016; Schilling et al. 2018). Recently, Teillac et al. (2017) proposed a method to improve the coverage of the bank of sulci using the pial surface information to guide the white matter reconstruction. Although further investigation is needed, the method shows promising results to reduce false negatives using anatomical information. Another drawback is the generation of false positives, i.e. the reconstruction of connections which are not really present (Maier-Hein et al. 2017). Methods to reduce false positives using microstructural properties of the white matter tissue are being proposed (e.g. Daducci et al. 2018). These use prior information on the tissue, such as volume, to remove streamlines not correctly representing it. Such methods have the potential to reduce the false positives problem of tractography but remain exploratory and preliminary. Also, since axon diameters remain constant along tracts (Innocenti et al. 2018), implementing biologically inspired tractography algorithm using diffusion MRI methods sensitive to axonal diameters (Assaf et al. 2008; Alexander et al. 2010; NODDI), will help us produce more accurate brain connectomes (Girard et al. 2017). The limitations of axonal diameter measurements using diffusion MRI are the source of heated debates beyond the aims of the present paper.

**Fig. 1:**
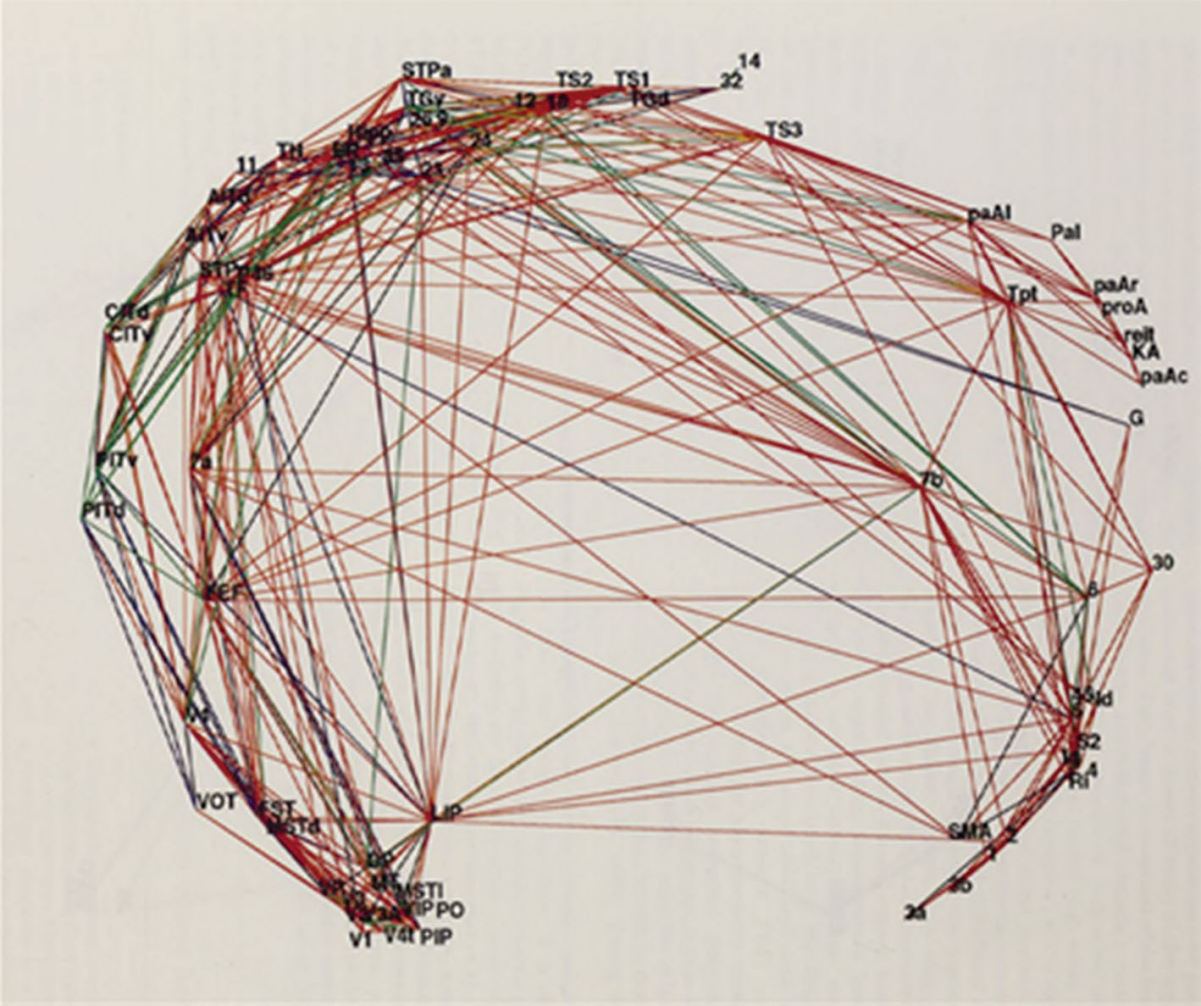
The topological organization of the entire macaque cortical processing system as then known. A total of 758 connections between the 73 areas are represented, of which 136 (18%) are one-way. This connectivity represents 15% of the possible connections between these areas. This non-arbitrary structure represents in a spatial framework the organizational structure of the network of cortico-cortical connections of this animal. For the explanation of symbols see Young (1993). An upgraded version of the same figure exists in Young et al. (1995) (from Young 1993, modified)

Topological principles underlying the organization of cortical connections could be used to further refine diffusion tractography and reduce false positives. Young (1992, 1993), Young et al. (1995); Fig. 1 spearheaded the attempt to produce cortical wiring diagrams by applying graph theory to the organization of cortical networks. His work and a later rich literature (Stephan et al. 2000; Chcklovskii et al. 2002; Klyachko and Stevens 2003; Markov et al. 2014; Wang and Clandinin 2016, among others) have established two principles of cortical wiring: (i) intercortical connections establish clusters of heavily interconnected areas, e.g., the somatomotor cluster, the visual cluster, the prefrontal cluster, etc. These clusters are characterized by massive connectivity between areas and are more sparsely connected with each other. Sparse connectivity is detected by specialized algorithms and might vary across individuals correlating with individual performance in neuropsychological tasks (Betzel et al. 2018). Therefore, it may not provide the ultimate mean for the refinement of tractography data. Nevertheless, in addition, (ii) Cortical connectivity obeys principles of economy, that is, it tends to minimize the volume occupied by axons. This principle was already stated by Cajal as: “loi de l’economie de protoplasma nerveux transmetteur et de temps de transmission” (law of the economy of neural protoplasm and of transmission times; Ramon y Cajal 1909) and was supported by other observations, among these the fact that gyration leads to economy of wiring (Innocenti 1990) and that the economy of wiring in evolution leads to limited increase in the diameter of cortical axons (Innocenti 2017) with consequential slowing down of cortico-cortical connectivity and increased dispersion of delays (Caminiti et al. 2009). It may also have led to relative loss of long connections as between mouse and monkey (Horvát et al. 2016). A third (iii) principle is that cortical areas with similar cytoarchitectonic features, essentially neuronal density, appear to be more frequently interconnected (Beul et al. 2017) while the distance between areas or cortical thickness are weaker predictors of connectivity.

The existence of functionally defined clusters can be used to accept or reject streamlines but with the limitations mentioned below. The principle of economy could also be used to eliminate streamlines whose length grossly deviates from the bulk of the others in the same bundle. Also, streamlines who grossly violate principles of economy in a diffusion MRI tractogram should be handled with skepticism.

The three principles mentioned above are rooted in developmental constraints and further advances in tractography for estimating brain connections might be achieved by exploiting some fundamental similarities between the diffusion tractography algorithms and those implemented in the development of neural connections. Below we list some of the similarities.

**Fig. 2:**
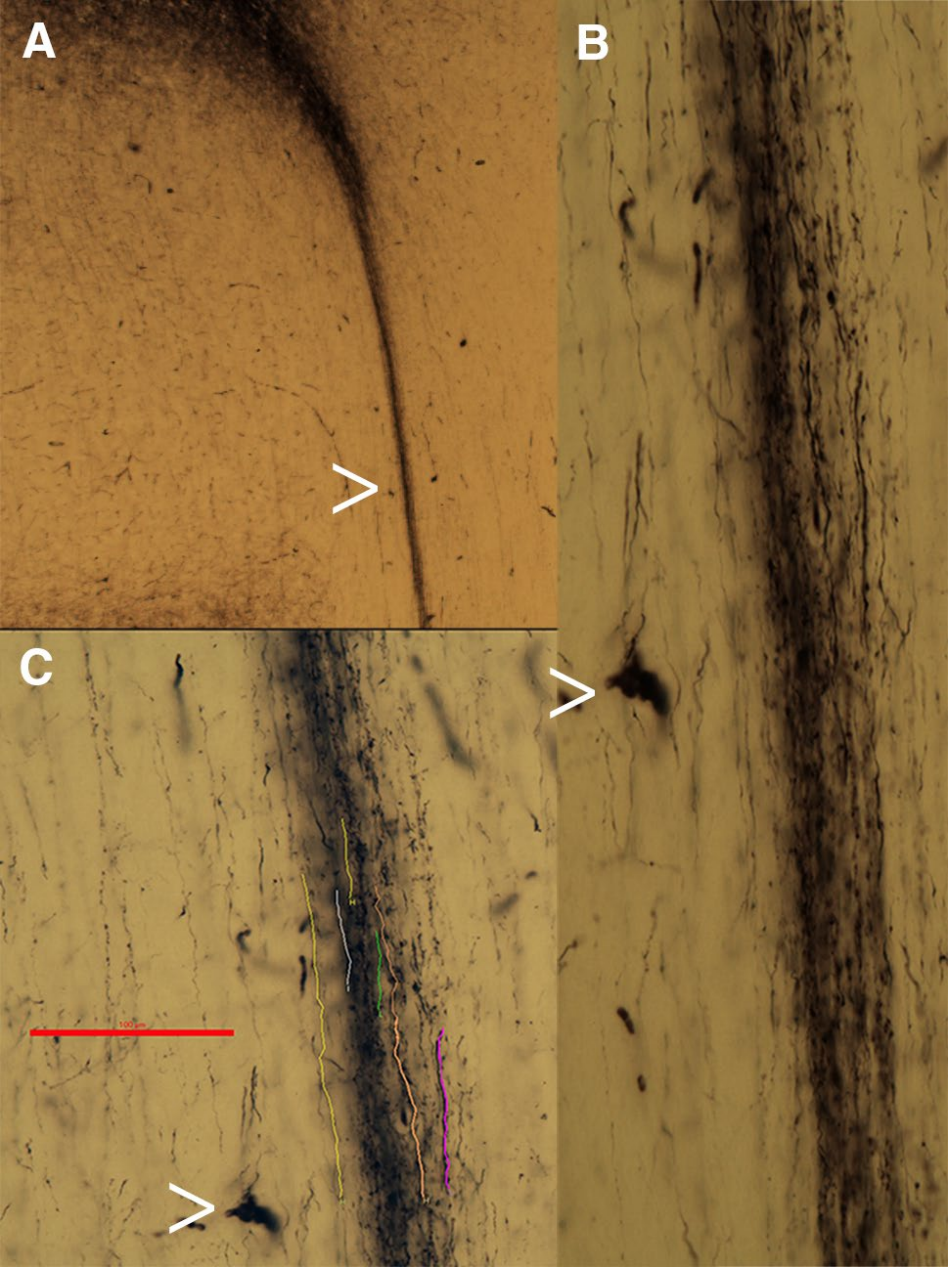
Axons are organized in tight fascicles in their initial trajectory. **a** Shows BDA labeled axons originating from an injection site near the areas 9/46 border in a macaque. **b, c** show enlarged views of the axonal fascicle. In **c** some axonal segments are down for clarity. The axons defasciculate further down along their course (see Fig. 1 in Caminiti et al. 2009)

**Fig. 3:**
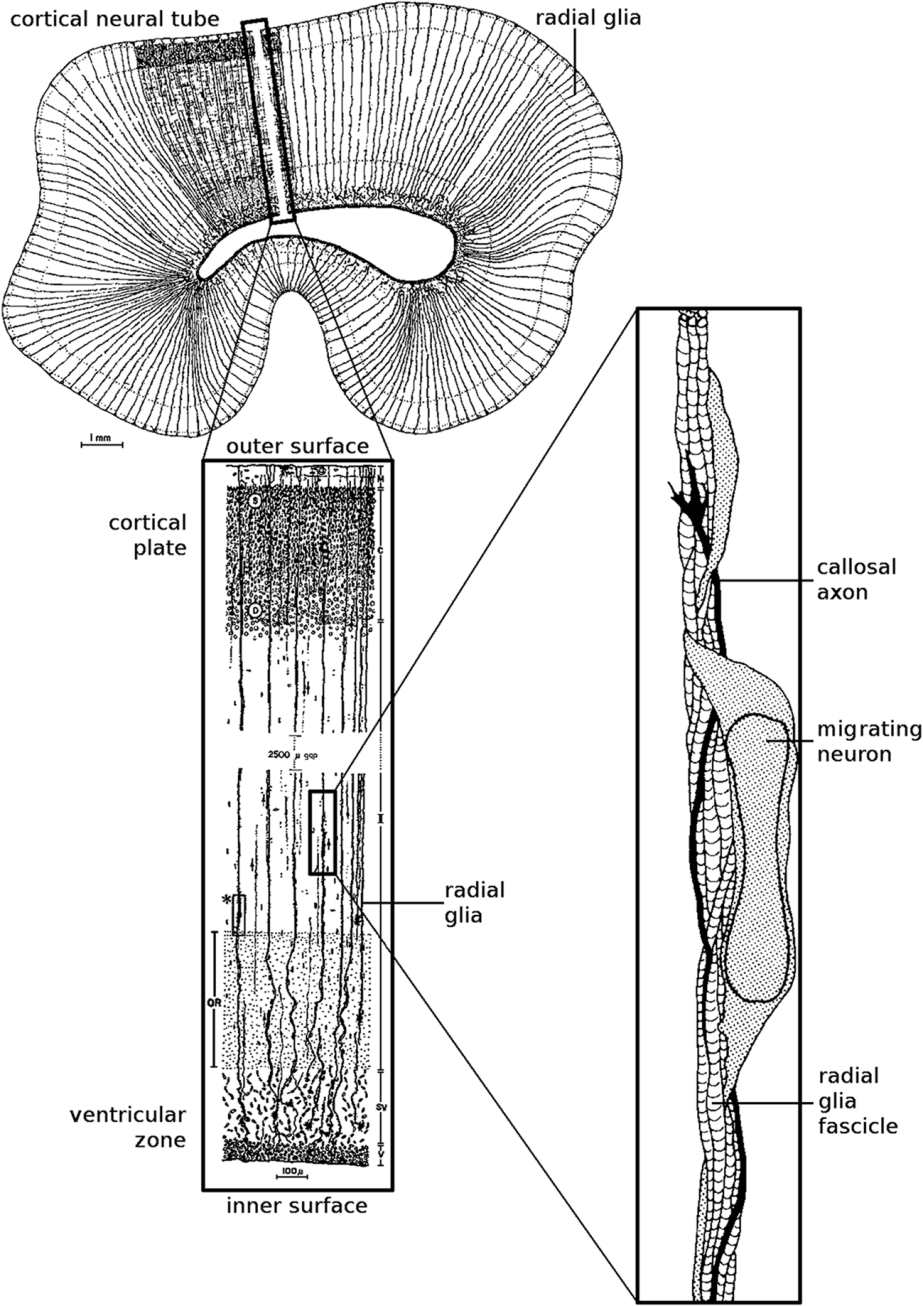
Schematic representation of radial glia at the time of neuronal migration and of axonal ingrowth. The left part of the figure is borrowed from Rakic (1972). The right part of the figure is reproduced from Norris and Kalil (1991)

- First. Axons tend to grow in tight fascicles in their initial trajectory (Fig. 2) although they can de-fasciculate further in their course when encountering other guiding cues (see Fig. 1 in Caminiti et al. 2009).
- Second. Neural connections develop when the brain is rather different from the adult. Growing axons navigate in the white matter led by attractive and repulsive cues (Kolodkin and Tessier-Lavigne 2011). At the time of axonal growth gyri and sulci have not formed yet and, when they do, they alter the already established trajectory of axonal fascicles.
- Third. The white matter is structured; it contains “guidepost” cells, pioneer axons, as well as glial fascicles (Rakic 1972), all of which orient the progress of growth cones (Norris and Kalil 1991; Fig. 3) and other axonal projections (Molnar et al. 1998) to which growing axons fasciculate.

Algorithms akin to development are already implemented in diffusion tractography.

First. Attracting and repulsive ROIs are usually inserted at chosen locations to guide the trajectories of streamlines. A good example is the MAGNET approach recently developed for better optic radiation reconstruction (Chamberland et al. 2017), or more recent bundle specific tractography (Rheault et al. 2017).

Second. Aberrant streamlines, whose trajectory deviates from the bulk of a given projection are eliminated by inspection or by algorithms which tend to preserve the bundling of axons (Côté et al. 2015; Prieto et al. 2016; Meesters et al. 2017). These algorithms also achieve economy of connections.

**Fig. 4:**
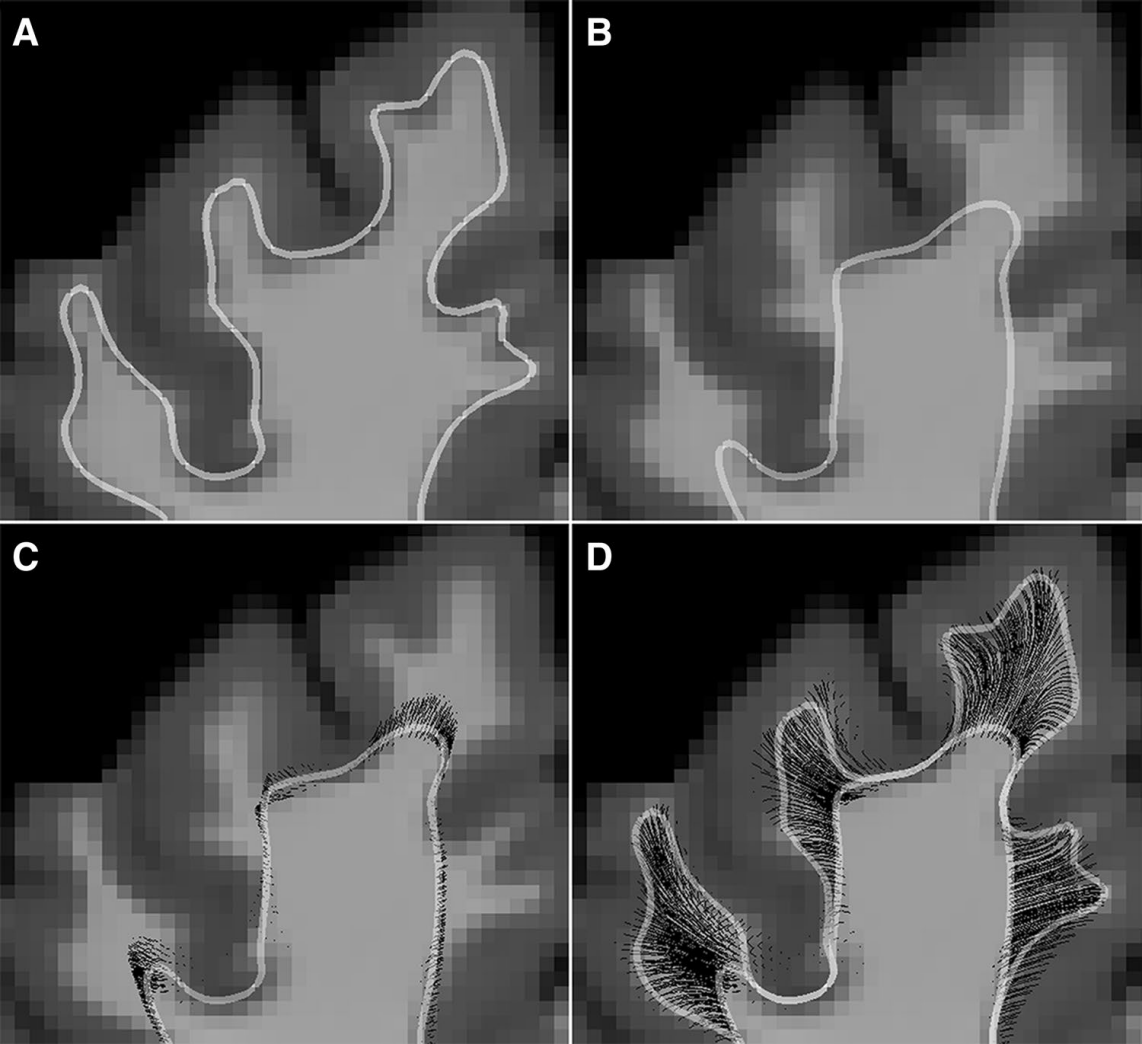
The cortical flow algorithm reproduces to some extent the growth of axons into the gray matter in early development. **a** Shows the initial gyration, **b** the gyration after applying the cortical flow algorithm, **c** is the initial stage of streamline ingrowth, **d** the final stage of streamline ingrowth. See also Online animation: cortical flow.gif

Third. The gyral bias can be corrected by implementing the “cortical flow” algorithm (Fig. 4). This approach is loosely related to previous attempts to modify cortical geometry in order to resolve cortical layers (Waehnert et al. 2014). Essentially it regresses the cortical geometry to when gyri and sulci have not fully formed yet, and to when axons are guided by radial glia (St-Onge and Descoteaux 2018, St-Onge et al. 2018).

## Further perspectives

In early development, axons grow directionally, from origin to target. In doing so, as mentioned above, they often follow pioneer axons who found their way using cellular and molecular cues in the substrate. Diffusion MRI could implement a similar strategy to guide streamlines in the white matter.

The elimination of false positives remains particularly challenging. Many transient (exuberant) projection form in development and are later eliminated (reviewed in Innocenti and Price 2005; Luo and O’Leary 2005). The selection of which axons will be maintained and which will be eliminated involves two sets of cues, axon-target recognition, probably due to molecular affinities (as for retinotectal projection) and activity, the lack of which leads to axonal elimination.

Indeed one can inform tractography with additional priors inspired by brain development. One possibility is that connections might conform to the molecular (genetic) heterogeneity of the brain, at least of the cortical mantle (Richiardi et al. 2015). This, in turn, could cause similarities in neuronal proliferation and migration, hence in cytoarchitectonics (Beul et al. 2017). Another is that connections should link functionally complementary brain sites, e.g. (Huntenburg et al. 2018). Functional criteria are at the basis of the clusterization of cortical areas, mentioned above. Two of us used functional criteria to accept the probable existence of an interhemispheric parieto-striatal connection in humans, which is less evident or absent in the monkey, but which might be involved in language (Innocenti et al. 2016). For sure, connections dealing with language are easier to accept in humans although they might be missing in other primates (Rilling et al. 2008).

Unfortunately, both molecular and functional criteria might lead to the rejection of connections inconsistent with *a priori* theoretical views, that is, the rejection of interesting, because unexpected, connections.

Eventually, in humans, coherent cortical activity revealed by EEG and MEG (Carmeli et al. 2005; Deslauriers-Gauthier et al. 2017) might provide the best tool to identify connected sites, particularly when the possibility that coherent activity might be generated by shared input, rather than by interconnections could be ruled out.

## Electronic supplementary material

Below is the link to the electronic supplementary material.

Supplementary material 1 (GIF 680 KB)
