# Supplementary figures and images for: Topological principles and developmental algorithms might refine diffusion tractography
Source: Brain Struct Funct. 2018 Sep 27;224(1):1–8. doi: 10.1007/s00429-018-1759-1 (PMC6373358; doi:10.1007/s00429-018-1759-1)

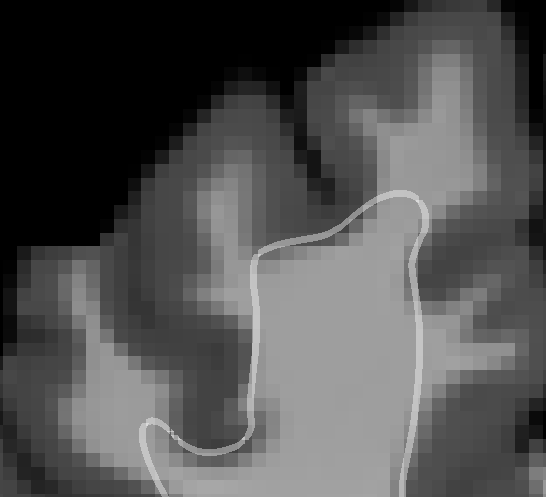

Supplement: Supplementary file 1 — Supplementary material 1 (GIF 680 KB) [file 429_2018_1759_MOESM1_ESM.gif]
